# Supplementary material for: Computational screening methodology identifies effective solvents for CO2 capture
Source: Commun Chem. 2022 Mar 18;5:37. doi: 10.1038/s42004-022-00654-y (PMC9814075; doi:10.1038/s42004-022-00654-y)
Supplement: Supplementary file 1 — Supplementary Information [file 42004_2022_654_MOESM1_ESM.pdf]

Supplementary Information

Computational screening methodology identifies effective solvents for CO_2 capture

Alexey A. Orlov[1], Alain Valtz[2], Christophe Coquelet[2], Xavier Rozanska[3], Erich Wimmer[3], Gilles Marcou[1], Dragos Horvath[1], Bénédicte Poulain[4], Alexandre Varnek[1], Frédérick de Meyer[2,4*].*

1 Laboratory of Chemoinformatics, Faculty of Chemistry, University of Strasbourg, 67081 Strasbourg, France

2 MINES ParisTech, PSL University, Centre of Thermodynamics of Processes (CTP), 35 rue St Honoré, 77300 Fontainebleau, France

3 Materials Design SARL, 42 avenue Verdier, 92120 Montrouge, France

4 TOTALEnergies S.E, OneTech, Gas & Low Carbon Entity, CCUS R&D Program, 2 Place Jean Millier, 92078 Paris, France

*corresponding author: frederick.de-meyer@totalenergies.com

Keywords: greenhouse gas, carbon dioxide, chemical solvents, tertiary amines, molecular simulations, chemoinformatics, machine learning

Supplementary Table 1. Absorption rate (R_{MD}, in g CO_2 L^{-1} min^{-1}), energy of absorption (ΔG_{MD}, in kJ mol^{-1}) predicted by molecular dynamic simulations and pK_a values, either the experimental ones or predicted by OPERA model.[1]

cas	smiles	Predicted values		
		R_{MD}	ΔG_{MD}	pKa
108-01-0	CN(C)CCO	3.38	-59.89	9.49
3179-63-3	CN(C)CCCO	2.91	-59.00	9.54
100-37-8	CCN(CC)CCO	4.83	-64.12	10.01
622-93-5	CCN(CC)CCCO	5.06	-65.56	10.29
108-16-7	CC(O)CN(C)C	4.17	-62.75	9.67
4402-32-8	CCN(CC)CC(C)O	3.50	-62.61	10.18
96-80-0	CC(C)N(CCO)C(C)C	2.00	-57.61	10.03
7005-47-2	CN(C)C(C)(C)CO	2.49	-60.13	10.34
19059-68-8	CN(C)CC(C)(C)CO	2.27	-57.77	9.54
5464-15-3	CCN(C)CCC(C)O	2.81	-59.49	9.82
139-87-7	CCN(CCO)CCO	1.50	-52.56	8.86
121-93-7	CC(C)N(CCO)CCO	2.59	-57.89	9.12
2160-93-2	CC(C)(C)N(CCO)CCO	3.69	-61.46	9.06
623-57-4	CN(C)CC(O)CO	2.12	-56.46	9.14
621-56-7	CCN(CC)CC(O)CO	7.00	-67.35	9.89
102-71-6	OCCN(CCO)CCO	1.48	-50.94	7.85
2955-88-6	OCCN1CCCC1	4.78	-63.29	9.86
85391-19-1	OCC(O)CN1CCCC1	2.63	-58.87	9.64

3040-44-6	OCCN1CCCCC1	4.21	-62.68	9.76
4847-93-2	OCC(O)CN1CCCCC1	6.60	-66.26	9.49
533-15-3	CN1CCCCC1CCO	6.08	-66.37	9.89
3554-74-3	CN1CCCC(O)C1	1.96	-55.50	8.94
13444-24-1	CCN1CCCC(O)C1	1.78	-54.95	9.21
105-59-9	CN(CCO)CCO	2.61	-57.30	8.65
693288-48-1	CCN(CC)CC(O)CCCO	4.64	-68.03	10.96
1009-65-0	CN(C)CC1CC(O)C(C)(C)O1	1.18	-50.78	8.38
118870-35-2	CN1CCC(C(O)CO)C1	3.73	-61.53	9.57
25727-94-0	CCCCCCCCCCN1CCOCC1	0.70	-44.45	7.52
437999-32-1	OCCN(CCO)C1CCCCC1O	2.08	-57.05	9.45
3018-41-5	CN(C)CCSCCN(C)C	2.44	-56.72	8.45
5842-09-1	CC(C)CN(CCS)CC(C)C	3.01	-62.80	10.41
254448-29-8	CN(CCOCCO)CCOCCO	1.24	-52.45	9.13
55675-72-4	CN1CCSCC1	1.50	-52.19	8.59
61168-09-0	CN(C)C1CCC(O)CC1	4.23	-63.22	9.95
854652-99-6	CN(C)CC(CO)(CO)CO	1.86	-56.10	9.42
90652-41-8	CSCCCCC(C(C)C)N1CCCC1	2.76	-59.57	9.35
629165-64-6	CCN1CC(O)CC(O)C1	3.52	-60.77	9.38

1920-46-3	CC(S)CN(C)C	2.46	-58.83	9.92
10295-91-7	CCN(C)C1COCC1O	2.29	-56.94	9.24
86375-49-7	CC(C)(C)CC(C)(C)N(CCO)CCO	4.25	-63.16	9.31
46321-78-2	CC(CO)N1CCN(C(C)CO)CC1	1.70	-53.71	8.42
55972-87-7	OCCCCN(CCCCO)CCCCO	1.54	-55.28	9.55
14548-72-2	CC(O)CN(CC(C)O)C1CCCC1	1.47	-54.59	9.53
857384-40-8	CCN1CCCCC1CCCO	6.09	-68.74	10.67
4223-94-3	CC(O)CN1CCN(C)CC1	4.72	-62.58	8.94
71002-70-5	CC(CCCO)N1CCOCC1	1.14	-50.38	8.40
90854-88-9	CCCN(CCC)CC(C)(C)CO	5.86	-67.05	10.19
6039-36-7	CC1CCCC(CO)N1CCO	6.66	-66.64	9.69
496-47-9	CC(O)CC1CCCN1C	4.46	-64.22	10.12
4134-00-3	CCC(O)CN(CC(C)O)CC(C)O	2.22	-57.70	9.23
34753-59-8	CC(C)N(CCCO)CCCO	2.59	-59.58	9.84
62237-08-5	CN(CCO)CCCN1CCCC1	3.95	-65.03	10.65
857832-28-1	CCOC(CC)CN(CCO)CCO	1.58	-53.09	8.25
102450-14-6	CC1(C)CCCN(CCO)C1	5.96	-68.32	10.61
2109-64-0	CCCCN(CCCC)CC(C)O	3.59	-64.20	10.45

1194736-09-8	CCCC(O)CCCN(C)C1CC CC1	1.70	-56.62	9.92
106694-61-5	OCCCN1CCCC(CO)C1	6.07	-66.74	10.08
6006-58-2	CCN(CC)CCSCCN(CC)C C	1.36	-52.85	8.73
14037-83-3	CN(CCO)CCN(C)CCO	1.22	-51.87	9.00
122-96-3	OCCN1CCN(CCO)CC1	2.58	-55.96	7.98
15433-27-9	OCC(CO)N1CCCCC1	4.23	-63.20	9.87
3574-43-4	CC(C)CCN(CCO)CCC(C) C	3.27	-63.33	10.35
91014-23-2	CCCCN(C)CCCCCO	2.48	-60.57	10.36
100-74-3	CCN1CCOCC1	1.28	-50.46	8.15
91425-90-0	CCN(CC)C1CCCC1O	3.40	-64.47	10.83
873376-29-5	CC(CO)CN1CCCC1	6.23	-67.23	10.23
13297-91-1	OCCN(CCO)CCCN1CCO CC1	0.69	-43.86	7.46
4500-30-5	OCCCN(CCCO)C1CCCC C1	2.13	-58.21	9.93
140-82-9	CCN(CC)CCOCCO	3.20	-60.40	9.43
1087704-96-8	CC(C)C(O)CCN1CCCCC 1	4.15	-65.22	10.47
30727-29-8	CN(C)C1CCCCC1O	5.75	-66.68	10.32

58883-81-1	CCN(CC)CCCN(CCO)CCO	1.32	-52.91	9.04
14400-71-6	CC1OC(CN(C)C)CC1O	1.36	-51.78	8.43
854464-00-9	CC(O)CCN(CCO)CCO	1.59	-54.02	8.77
872816-90-5	CC1CCCCN1CCCCCCO	1.56	-56.01	10.07
4402-30-6	CC(O)CN(C)CC(C)O	1.16	-51.16	8.75
2842-41-3	CN(CCO)C1CCCCC1	3.51	-62.77	10.30
3492-47-5	CCN(CC)CC(O)CN(CC)CC	1.88	-57.33	9.90
854665-75-1	OCCCSCCN1CCOCC1	0.69	-38.39	6.25
59941-28-5	CCCCCCCC1CCCN1CCCO	1.36	-52.85	8.65
1039627-29-6	CCC(O)CCCN1CCCC1	3.41	-62.21	10.08
144205-48-1	CCCN(CCCO)CCCO	2.83	-59.61	9.43
4667-57-6	SCCCCCN1CCCCC1	0.98	-49.55	8.47
110513-60-5	CC(C)(O)CCN1CCCCC1	5.57	-67.32	10.44
15520-05-5	CCCCCCCCN(CCO)CCO	0.71	-45.96	7.95
42434-24-2	CN(CCCO)CCCN(C)CCCO	1.13	-52.49	9.53
106694-59-1	CN(CCO)CCCO	1.91	-55.45	9.03
2421-02-5	CCC(O)CN(CC(O)CC)CC(O)CC	1.79	-56.21	9.25
4767-14-0	OCCCN(CCO)CCO	1.71	-53.40	8.18

4402-34-0	CCCCN(CC(C)O)CC(C)O	2.04	-56.93	9.29
10315-98-7	CC(C)CN1CCOCC1	0.85	-47.45	8.16
934-90-7	CC(O)CN1CCCCC1	4.45	-64.23	10.18
94473-23-1	CCCCN(CCCC)CCCCO	1.86	-58.28	10.23
854815-53-5	CN(CCO)C1CCCCC1O	2.68	-59.06	9.57
104-58-5	OCCCN1CCCCC1	3.80	-63.41	10.33
622-40-2	OCCN1CCOCC1	0.64	-42.39	7.28
70787-41-6	CC(C)(CO)N(CCO)CCO	2.30	-57.25	8.96
82718-59-0	CN(C)CC(CO)(CO)CN(C)C	2.60	-58.55	9.18
106694-60-4	OCCCN1CCCCC1CCO	5.42	-67.27	10.52
1005-67-0	CCCCN1CCOCC1	1.30	-50.91	8.20
6735-35-9	CCCN(CCO)CCO	1.57	-53.39	8.66
20473-84-1	CCN(CC)CCCC(O)CO	4.18	-66.49	10.82
57567-83-6	CN(C)CCCN(CCO)CCO	1.58	-54.05	8.95
20966-69-2	CN(C)CCN1CCN(CCO)CC1	4.06	-61.02	8.63
4344-62-1	OCCCCCN1CCOCC1	1.30	-51.66	8.55
98956-90-2	OCCN(CCO)C1CCCC1	3.25	-60.40	9.41
10353-86-3	CC(O)CN(CCO)CC(C)O	1.19	-51.18	8.50
21461-57-4	CCN(CCO)C1CCCCC1	4.82	-65.95	10.43
6852-28-4	CCN(CC(C)O)CC(C)O	2.54	-58.12	9.06
14002-34-7	OCCCN(CCCO)CCCO	1.99	-56.53	9.21

6712-98-7	CC(O)CN(CCO)CCO	1.02	-49.67	8.43
2109-66-2	CC(O)CN1CCOCC1	0.78	-44.01	7.26
6289-52-7	CCCCN(CCCC)CC(O)CO	3.04	-61.10	9.76
115188-50-6	CN(CCO)CCOCCN(C)CCO	0.90	-49.38	8.81
4711-14-2	OCCCCCCN1CCCCC1	3.12	-61.99	10.26
122-20-3	CC(O)CN(CC(C)O)CC(C)O	2.28	-57.34	8.90
59941-12-7	CCCCCC1CCCN1CCO	2.42	-57.64	8.98
64897-89-8	CC(C)CCN(CCO)CCO	2.33	-57.59	9.13
40694-17-5	CN(CCO)CCCCCO	2.40	-58.82	9.90
2160-93-2	CCCCN(CCO)CCCC	3.55	-63.06	10.20
100-37-8	CN(CCCO)CCCO	3.07	-59.82	9.31
139-87-7	CCCCN(CCS)CCCC	4.91	-70.36	11.24
4847-93-2	CCCN(CCS)CCS	2.58	-60.39	10.18
3040-44-6	CC(CS)CN1CCCC1	3.23	-62.42	10.36

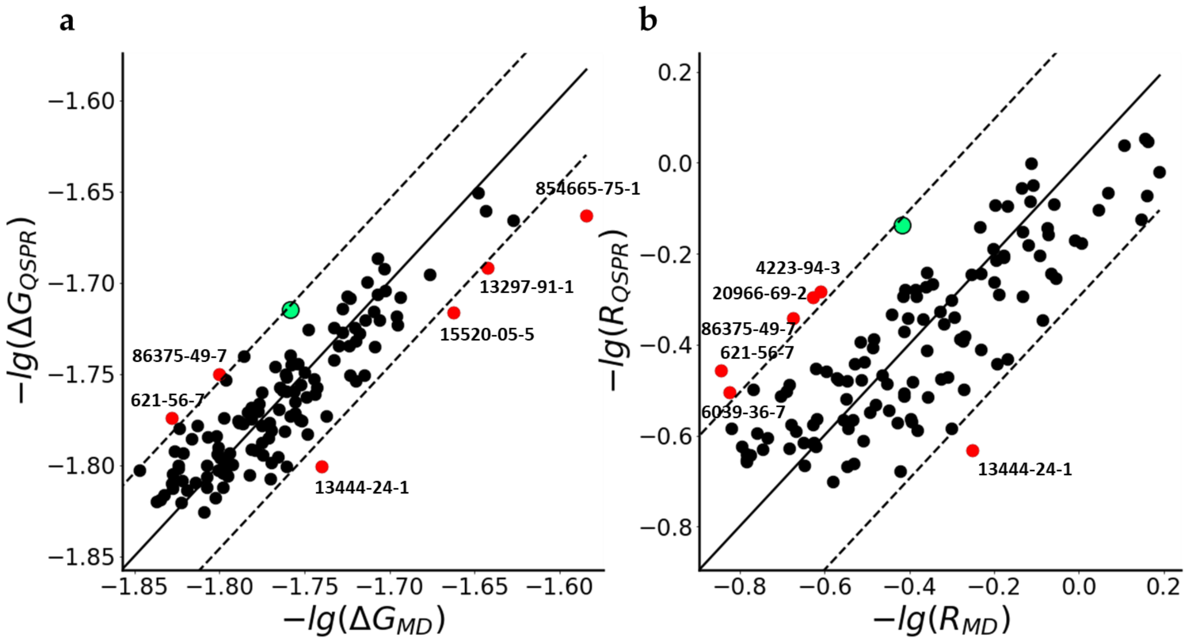

Supplementary Figure 1. a, -log$_{10}$(ΔG_{MD}) values vs the absorption rates predicted in the cross-validation procedure using QSPR (-lg(ΔG_{QSPR})) **b**, -log$_{10}$(R_{MD}) values vs the absorption rates predicted in the cross-validation procedure using QSPR (-log$_{10}$(R_{QSPR})). Compounds (with CAS numbers) for which absolute errors were larger than 2×RMSECV are shown in red. Dash lines indicate ±2×RMSECV threshold. Industrially used reference compound (MDEA) is shown in green.

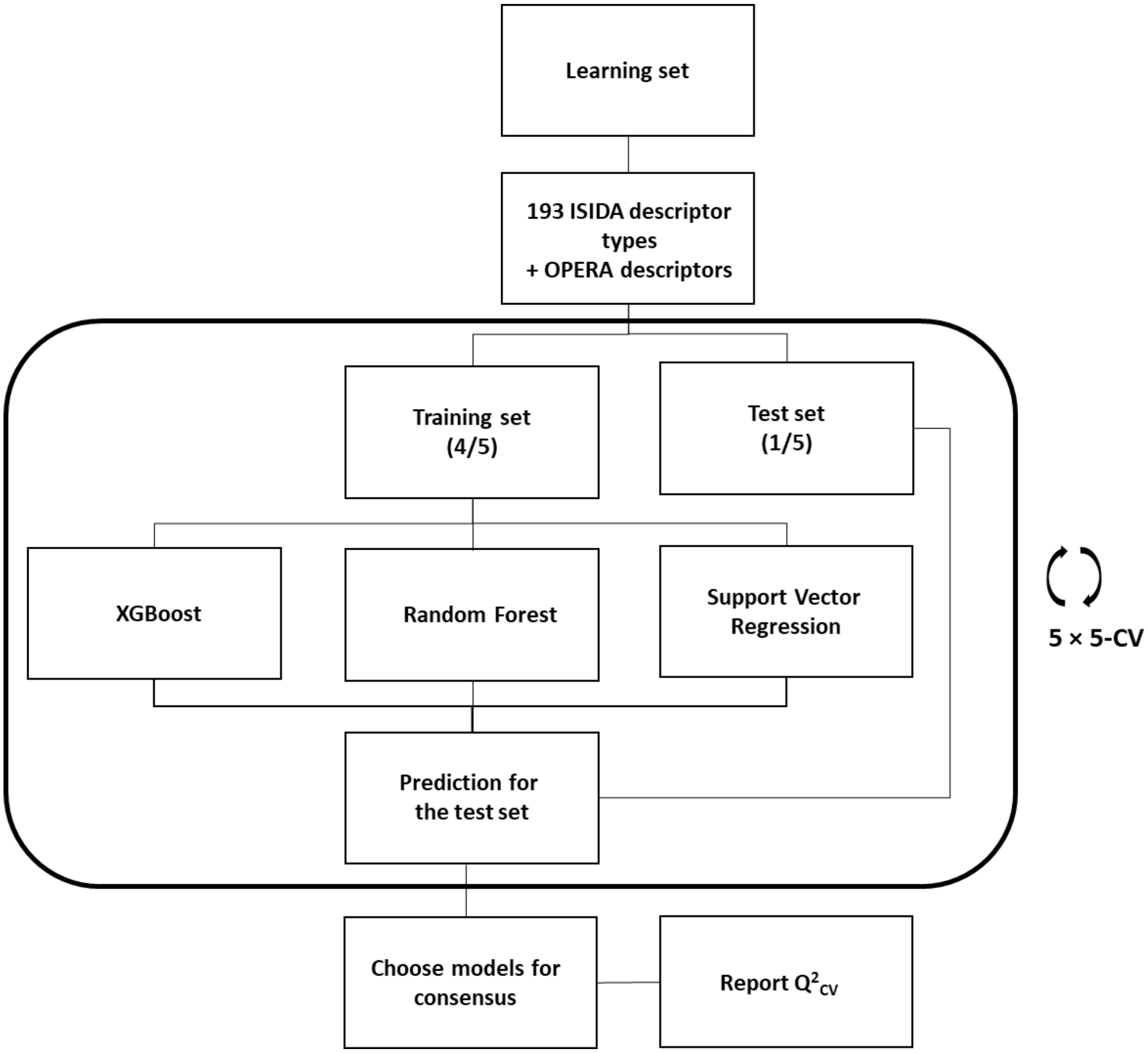

Supplementary Figure 2. A scheme for building the ISIDA consensus model. For the structures from the learning set 193 ISIDA fragment types and OPERA descriptors were generated. Each ISIDA descriptor space was combined with OPERA descriptors. Five-fold cross-validation was then used to assess the prediction performance of models. The cross-validation was repeated 5 times. For Spartan descriptors the models were built in the same way.

Supplementary Table 2. Performance estimation for modeling in 5 times repeated 5-fold cross validation.

End-point	Models	5x5 repeated cross-validation			5x5 nested cross-validation		
		Q^2_{CV}	$RMSE_{CV}$	MAE_{CV}	Q^2_{NCV}	$RMSE_{NCV}$	MAE_{NCV}
R_{MD}	18 Random Forest models + 18 XGBoost models built on ISIDA + OPERA descriptors	0.65±0.03	0.15±0.01	0.12±0.01	0.64±0.03	0.15±0.01	0.12±0.01
	(baseline model) Linear regression built on predicted pKa values	0.57±0.01	0.16±0.01	0.13±0.01	-	-	-
ΔG_{MD}	16 Random Forest models + 12 XGBoost models built on ISIDA + OPERA descriptors	0.77±0.02	0.02±0.01	0.02±0.01	0.77±0.01	0.02±0.01	0.02±0.01
	(baseline model) linear regression built on predicted pKa values	0.78±0.02	0.02±0.01	0.02±0.01	-	-	-

Supplementary Table 3. Purity of the purchased compounds.

Name	Abbrv.	CAS No.	MW (g/mol)	Purity %	Supplier	Bp
1-Ethyl-3-hydroxypiperidine	EHP	13444-24-1	129.20	98%	TCI	
2-(2-(dimethylamino)ethoxy)ethanol	DMAEE	1704-62-7	133.19	98%	Aldrich	95 °C(15mmHg)
n-Methyl-3-piperidinol	MPOL	3554-74-3	115.17	98.8%	Apollo Scien.	76-78 °C(11mmHg)
3-Piperidino-1,2-propanediol	PPOL	4847-93-2	159.23	96%	Aldrich	77-80 °C
n-Methyldiisopropanolamine	MDIPA	4402-30-6	147.215	95%	Ambinter	228.35 °C
1-Methyl-4-piperidinemethanol	MPM	20691-89-8	129.20	97%	TCI	108 °C (8mmHg)
4-Hydroxy-1-methylpiperidine	HMP	106-52-5	115.18	98%	TCI	105 °C (2.4KPa)
1-Methyl-3-pyrrolidinol	1MPOL	13220-33-2	101.15	97%	TCI	75 °C (1.6KPa)
1-Ethyl-3-pyrrolidinol	EPOL	30727-14-1	115.18	95%	TCI	110 °C (4.7KPa)
Methyldiethanolamine	MDEA	105-59-9	119.16	99 + / GC	Aldrich	243 °C
N-Ethyldiethanolamine	EDEA	139-87-7	133.19	98%	Sigma-Aldrich	246 – 252 °C
2-[2-‘Diethylamino) ethoxy] – ethanol	DEAE-OH	140-82-9	161.25	98	TCI	101 °C (9.8 mmHg)
1-[Bis(2-hydroxyethyl)amino]-2-propanol	HAP	6712-98-7	163.22	93%	TCI	145 °C (0.6mmHg)
Triethanolamine	TEA	102-71-6	149.19	99.8%	VWR	190-193 °C (7hPa)
3-(Diethylamino)-1, 2-propanediol	DIAP	621-56-7	147.22	98%	TCI	233 °C
Tetrakis(2-hydroxyethyl)ethylenediamine	THEE	140-07-8	236	97%	Aldrich	280 °C
N-Methylmorpholine	NMM	109-02-4	101.25	99.5%	Acros	116 °C
Tetrakis(2-Hydroxypropyl)ethylenediamine	THPE	102-60-3	192.41	98%	Aldrich	175-181 °C (0.8mmHg)
3-Dimethylamino-1-propanol	DMAP	3179-63-3	103.16	99%	Aldrich	163 °C
Piperazine	PZ	110-85-0	86.14	99%	Aldrich	146 °C

Supplementary Table 4. Slope r(CO_2) and time required to absorb 50% of CO_2 (t^{50}_{CO2}, s).

cas	smiles	r(CO_2) (s^{-1})	t^{50}_{CO2} (s)
105-59-9	CN(CCO)CCO	0.00080	357
102-71-6	C(CO)N(CCO)CCO	0.00030	1226
109-02-4	CN1CCOCC1	0.00020	1260
6712-98-7	CC(CN(CCO)CCO)O	0.00023	1435
621-56-7	CCN(CC)CC(CO)O	0.00129	153
140-82-9	CCN(CC)CCOCCO	0.00084	283
140-07-8	C(CN(CCO)CCO)N(CCO)CCO	0.00025	1677
139-87-7	CCN(CCO)CCO	0.00060	597
102-60-3	CC(CN(CCN(CC(C)O)CC(C)O)CC(C)O)O	0.00037	2105
13444-24-1	CCN1CCCC(C1)O	0.00060	459
1704-62-7	CN(C)CCOCCO	0.00090	360
3554-74-3	CN1CCCC(C1)O	0.00070	406
4847-93-2	C1CCN(CC1)CC(CO)O	0.00063	660
20691-89-8	CN1CCC(CC1)CO	0.00110	249
4402-30-6	CC(CN(C)CC(C)O)O	0.00057	473
13220-33-2	CN1CCC(C1)O	0.00162	146
30727-14-1	CCN1CCC(C1)O	0.00200	147
106-52-5	CN1CCC(CC1)O	0.00133	278
3179-63-3	CN(C)CCCO	0.00193	83

Supplementary Methods.

The method for the calculation of the solubility of CO_2 is inspired form the “static-synthetic method”. The solubility is obtained after solving a system of equations taking into account the quantities introduced into the equilibrium cell, and the measurements of volume, temperature and pressure. The following describes the main principles of the “static synthetic method”.

Description of experimental set-up

A “static-synthetic” technique based on a closed-circuit method is used for the determination of acid gas solubility in the different solvents. The equilibrium cell is equipped with pressure transducers. Temperature is given by two platinum probes located at the upper and lower flanges (possibility to determine the gradient of temperature). An internal stirring system with external motor reduced the time required to reach equilibrium. Supplementary Figures 3 and 4 present the flowsheet of the equipment considered for the “static-synthetic “method. Supplementary Figure 4 was specially developed for measurement at low pressure (below 5 bar).

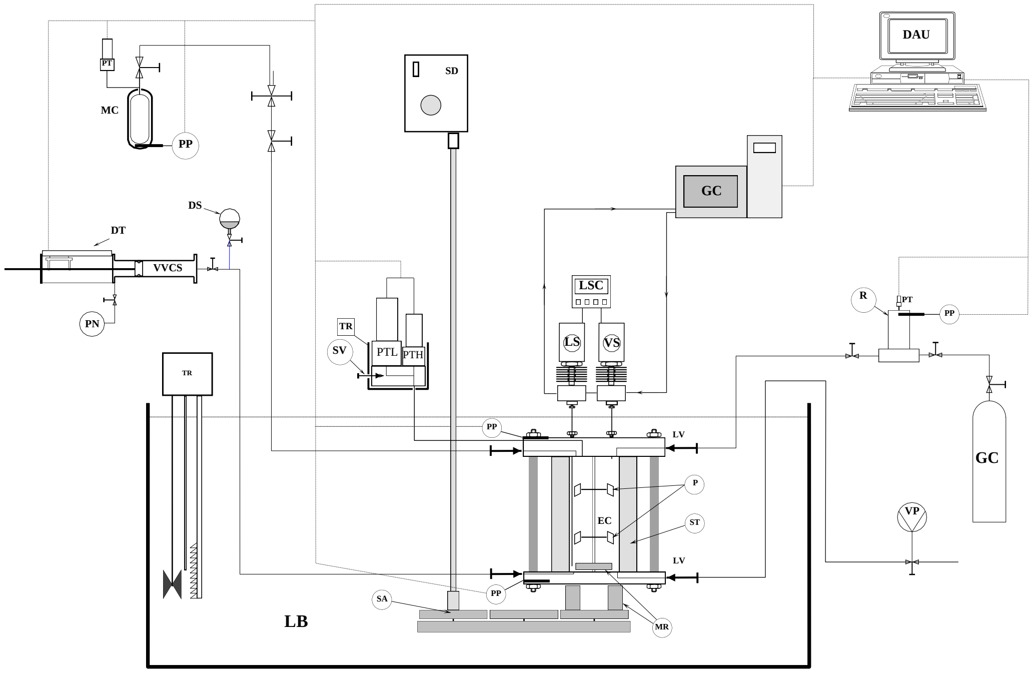

Supplementary Figure 3: Schematic diagram of apparatus: DS: Degassed Solution; d. a. u. : Data Acquisition Unit ; DS : Degassed Solution ; DT : Displacement Transducer ; EC : Equilibrium Cell ; GC : Gas Chromatograph ; LB: Liquid Bath; LS : Liquid Sampler ; LVi : Loading Valve ; MR : Magnetic Rod; P: Propeller; PN: Pressurized nitrogen; PP : Platinum Probe ; PTh: Pressure transducer for high pressure values; PTl: Pressure transducer for low pressure values; PT: pressure transducer ; R : Reserve ; SD : Stirring Device ; SM: Sample Monitoring; ST: Saphire tube; Th: Thermocouple; TR: Thermal Regulator; Vi: Valve; VP: Vacuum Pump; VS: Vapor Sampler; VVCM: Variable Volume Cell for Solution

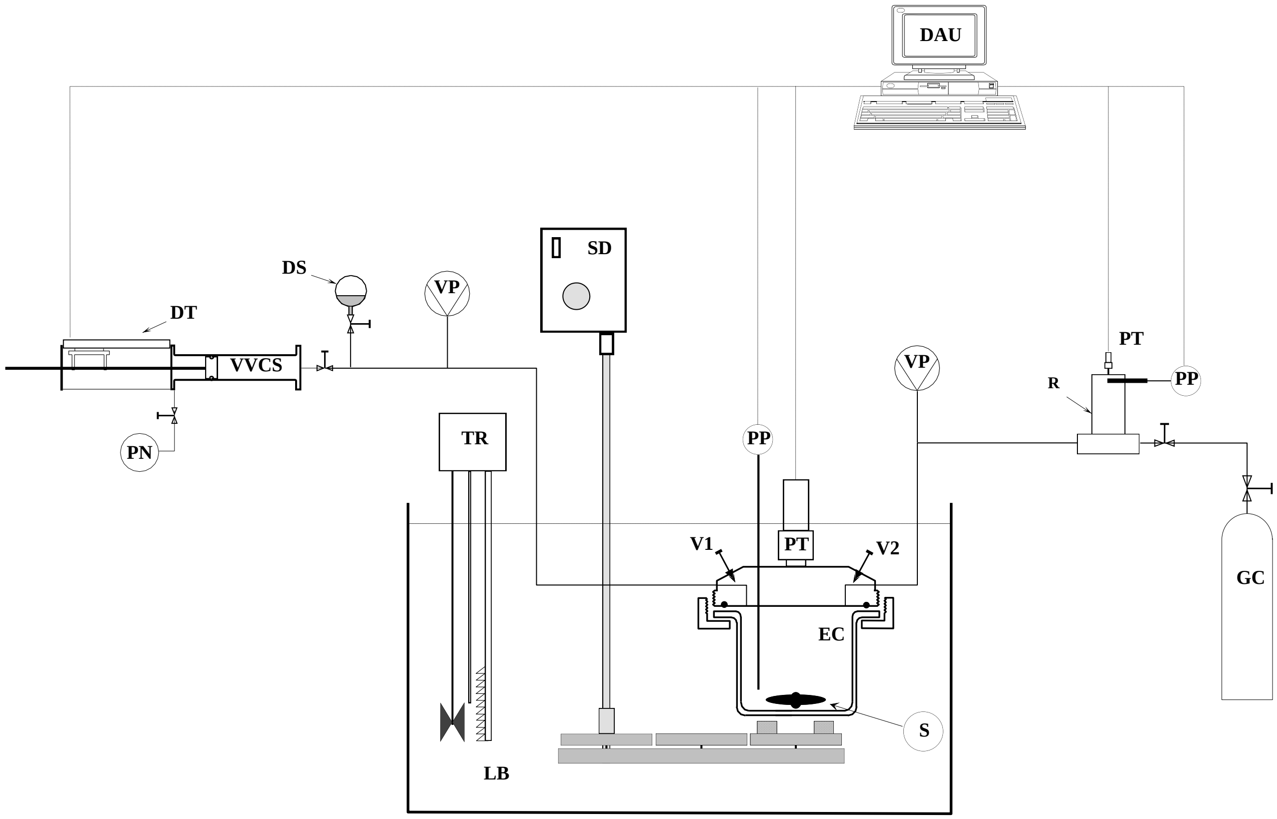

Supplementary Figure 4: Flow diagram of the synthetic apparatus: DAU: data acquisition unit; DS: degassed solution; DT: displacement transducer; EC: equilibrium cell; GC: gas cylinder; LB: liquid bath; PN: pressurized nitrogen; PP: Platinum probe; PT: pressure transducer; R: gas reservoir; S: stirrer; SD: stirring device; TR: thermal regulator; Vi: valve; VP: vacuum pump; VVCS: variable volume cell for solution.

In case of mixture, the vapor phase is analysed. The apparatus is equipped with at least one online capillary sampler (ROLSI®, Armines' patent) which are capable of withdrawing and sending micro samples to a gas chromatograph without perturbing the equilibrium conditions over numerous samplings, thus leading to repeatable and reliable results. Analytical work was carried out using a gas chromatograph (PERICHROM model PR2100, France) equipped with a thermal conductivity

detector (TCD) connected to a data software system. Helium is used as the carrier gas in this experiment. The model of the GC column is Porapak R (Porapak R 80 / 100 mesh, 1 m x 2 mm ID Silcosteel).

a. Calibrations of temperature sensors

The equilibrium cell temperature is measured at two points, one for vapor and the other for liquid phase by using two four-wire Pt100 Platinum Resistance Thermometer probes, which were calibrated against a reference four-wire PT-25 Platinum Resistance Thermometer probe (PT-100). The 25 Ω reference platinum resistance thermometer (TINSLEY Precision Instruments) was calibrated by the Laboratoire National d'Essais (Paris) based on the 1990 International Temperature Scale (ITS 90). The results accuracy for temperature is not higher than ± 0.03 °C by the temperature range.

b. Calibrations of pressure sensors

The pressure transducers have been calibrated against a PACE 5000 Modular Pressure Calibrator (GE Sensing France) for the high-pressure transducer and a numerical standard for the low-pressure transducer. The accuracy of the pressure transducer is ±0.0002 MPa for the high-pressure transducer and ±0.0001 MPa for the low-pressure transducer. For the low-pressure equipment, a flush diaphragm pressure transducer (P_{max}=5 bar) is used with an accuracy of ±4.2 mbar.

c. TCD and FID Calibration Summary

GC syringe are used for the calibration of GC detectors. Estimation of the uncertainties are obtained with the help of polynomial expressions whereby the numbers of moles are expressed as a function of peak areas, which allow estimation of the uncertainties on measured numbers of moles through GC analyses. Supplementary Table 5 shows the calibrations of the GC detectors results.

Supplementary Table 5: Components and accuracy (example of typical accuracy values).

Component	Phase	Moles numbers	Detector (sensitivity)	Accuracy/ %
CH_4	Vapor	2.4639x10^{-7} - 2.0532x10^{-6}	TCD (gain=0.5)	2.5
CO_2	vapor	4.1691x10^{-7} - 4.1691x10^{-6}	TCD (gain=0.5)	2

1. Loading of the solvent

The solvent is introduced inside the equilibrium cell by a variable volume cell or a liquid syringe with a relative accuracy of 2%. In order to calculate the mole number of solvent introduced it is important to know its density.

Consequently, with a syringe Supplementary Equation (1) is applied,

$$n_{solvent} = \rho(T)V \quad (1)$$

where V is the volume introduced into the equilibrium cell.

With a variable volume cell, Supplementary Equation (2) is used

$$n_{solvent} = \rho(T)\,\pi\, r^2 \Delta l \quad (2)$$

where Δl is the piston displacement (accuracy ±0.01 mm), r the radius of the piston.

2. Loading press for CO_2 gas

The loading of acid gas CO_2 into the equilibrium cell is carried out through a reservoir having well known volumes (V). The arrangement is shown in Supplementary Figure 5. The pressure and temperature are continuously registered through data acquisition unit. Pressure inside the reservoirs are measured by means of Druck™ pressure transducers connected to the data acquisition unit (HP34970A). The temperature of the press is measured through one thermocouple which are also connected to the HP data acquisition unit.

Total mole number of solute introduced into the cell is given by considering indication from reservoir press. This value is calculated by considering the variation of T and difference of pressure during the loading (before and after the loading). Supplementary Equation (3) gives the equation to calculate the loading of CO_2 with ρ the density of the acid gas calculated using REFPROP v10.0 and V^{press} the volume of the press.

$$L_{CO2} = \frac{n_{CO2}^{before} - n_{CO2}^{after}}{n_{amine}} = V^{press}\,\frac{\rho(T^{before}, P^{before}) - \rho(T^{after}, P^{after})}{n_{amine}} \quad (3)$$

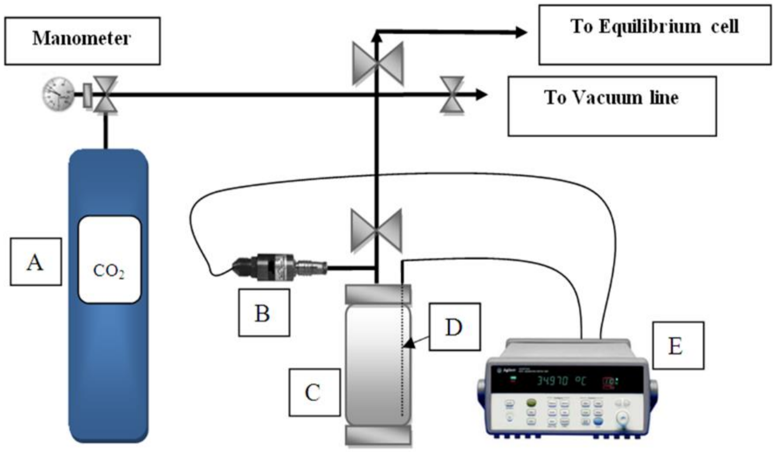

Supplementary Figure 5: A pictorial view of CO_2 loading arrangement, where A; Gas Cylinder CO_2 B; Pressure transducer, C; Reservoir press, D; Thermocouple, E; Data acquisition unit HP34970A.

3. Determination of the solubility

The calculation the acid gas solubility in the solvent is based on mass balance. The volume of liquid phase is obtained by considering the mole number of solvent introduced and its density at the temperature of measurement (Supplementary Equation (4)).

$$V^L = \frac{n_{solvent}}{\rho(T_{cell})} \quad (4)$$

Consequently, the volume of the vapor phase is calculated by difference from the total volume and the volume of the liquid phase considering Supplementary Equation (5).

$$V^V = V^T - V^L \quad (5)$$

If the introduction of the solute doesn't modify the level of the liquid interface in the equilibrium cell, we can consider Supplementary Equation (6).

$$V^L = \pi r_{cell}^2 h_{liq} \tag{6}$$

Where r_{cell} is the radius of the equilibrium cell, h_{liq} the level of the vapor liquid interface.

The mole number of solute in the vapor phase is calculated by considering the density of the gas at the temperature and pressure of solute ($P_{Solute} = P_{cell} - P_{solvent}^{sat}$). REFPROP v10.0 is used to calculate this density $\rho^V(T_{cell}, P_{solute})$. In case of mixture, the global composition have to be considered $\rho^V(T_{cell}, P_{solute}, y)$.

The volume of the vapor phase is used to calculate the mole number of solute in the vapor phase (Supplementary Equation (7)).

$$n^V = V^V \rho^V(T_{cell}, P_{solute}) \tag{7}$$

In case of mixture, the same equation is used to calculate the total mole number of solute in the vapor phase.

So, the mole number of solute in the liquid phase is determined by considering Supplementary Equation (8).

$$n^L = n^T - n^V \tag{8}$$

In case of mixture, the mole number of each species is calculated by considering the global composition of the mixture (z) and the composition of the vapor phase (y) is given by Supplementary Equation (9).

$$n_i^L = z_i n^T - y_i n^V \tag{9}$$

The solubility is determined with Supplementary Equation (10).

$$x_i = \frac{n_i}{\sum n_j} \tag{10}$$

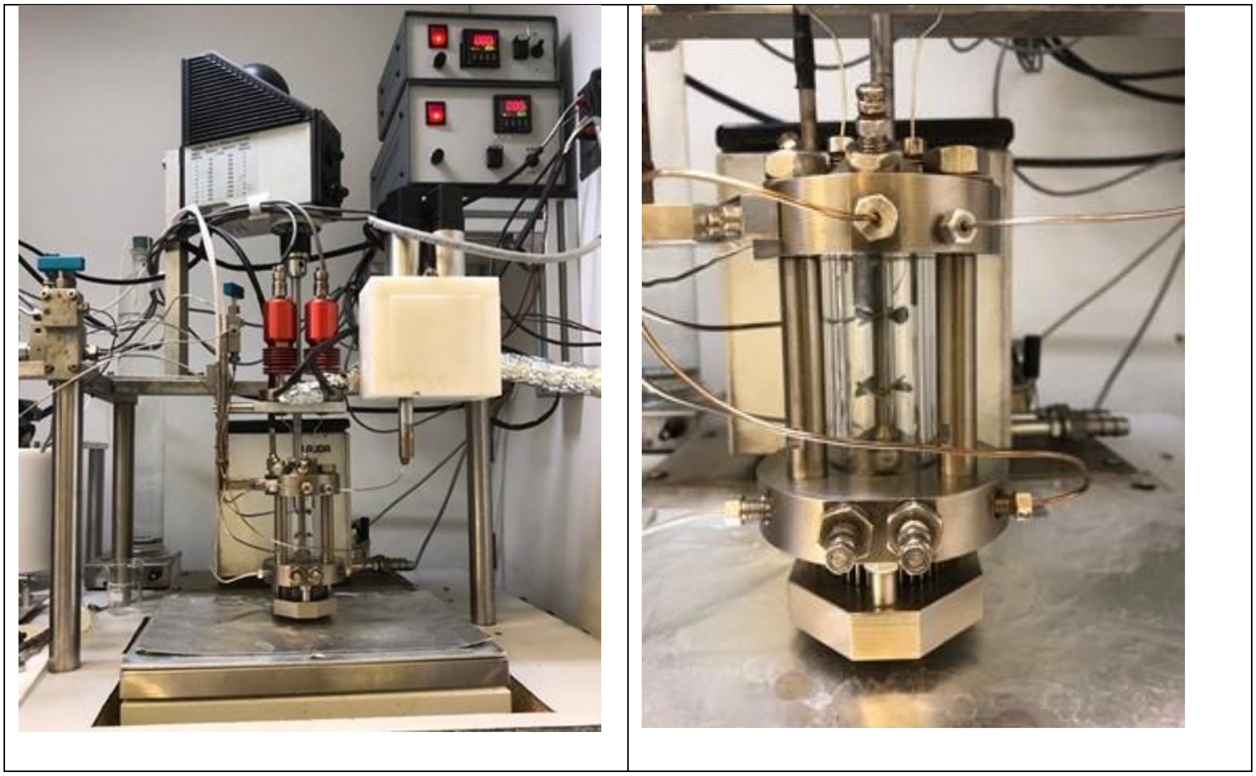

Supplementary Figure 6: Views of the experimental setup for solvent kinetic screening.

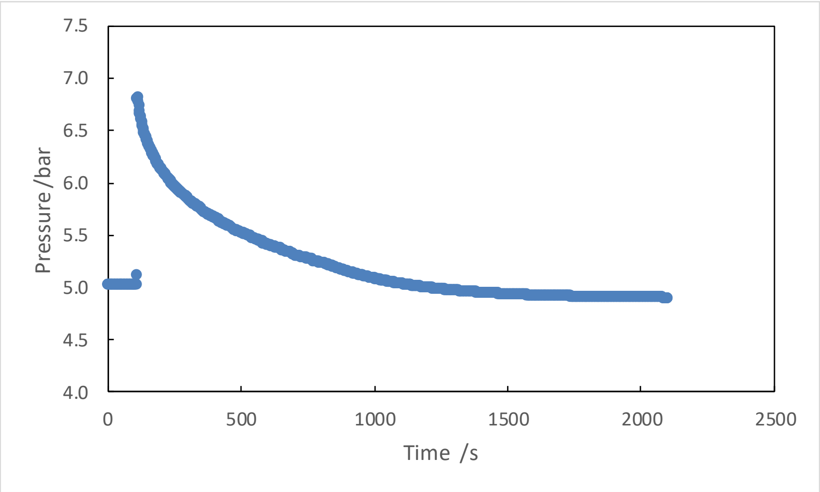

Supplementary Figure 7: Variation of total pressure with time.

4. Uncertainty calculations

There are two main sources of uncertainties: uncertainties due to calibration and uncertainty due to repeatability. The uncertainty due to calibration is of Type B. It means that after calibration we do not directly get the uncertainty but the accuracy. A statistic distribution needs to be chosen. In terms of probabilities, it is common to consider a rectangular statistic distribution. The rectangular distribution is given as $u_{calib}(\theta)=\frac{b}{\sqrt{3}}$. b is defined as the half-width between the upper and lower error limits (so the value of the accuracy). This value is obtained after polynomial regression between value given by the sensor and the reference value. For a normal distribution, ± u

encompasses about 68 % of the distribution; for a rectangular distribution, ± u encompasses about 58 % of the distribution.

During an experiment, to determine the value of one quantity (T, P or mole numbers) we have to consider all values acquired. It follows a calculation of average values and standard deviation δ. The averaging of repeated readings yields a mean θ_{avg} with a standard deviation δ. As before, this can be statistically converted to an uncertainty due to repeatability of the measurements, via Supplementary Equation (11).

$$u_{rep}(\theta) = \frac{\delta}{\sqrt{n}} = \sqrt{\frac{1}{n(n-1)}\sum_{i=1}^{n}\left(\theta_i - \theta_{avg}\right)^2} \qquad (11)$$

where, $\theta_{avg} = \frac{1}{n}\sum_{i=1}^{n}\theta_i$ and n is the number of repeated quantity measurements. A Gaussian type of distribution is the likely behavior here, since the repeated readings are likely to fall close to the mean (with maybe one or two values falling from the mean). This is known as a type A evaluation (systematic uncertainty), where only statistical methods are required to interpret the uncertainty. Supplementary Equation (12) presents the combined standard temperature uncertainty, u_c (T) with subscripts calib, rep denoting that of calibration, repeatability.

$$u_c(\theta) = \pm\sqrt{u_{calib}(\theta)^2 + u_{rep}(\theta)^2} \qquad (12)$$

The uncertainty of the composition include the purity of the chemicals used. It is calculated by the Supplementary Equation 13.

u_{purity} (m) is equal to (1-p)/$\sqrt{3}$ assuming uniform distribution with p the purity (example 99% purity leads to 1-p=0.01).

a. Uncertainty on mole fraction

The determination of the uncertainty of the composition required the uncertainty of each mole numbers. The uncertainty of the mole fraction is determined after calibration of the GC detectors Supplementary Equation (13).

$$u(x_i) = \sqrt{\sum_i^{ncomp} \left(\frac{\partial x_i}{\partial n_i}\right)^2_{i \neq j} u^2(n_i)} \qquad (13)$$

For a binary system, one can calculate $u(x_1) = x_1(1 - x_1)\sqrt{\left(\frac{u(n_1)}{n_1}\right)^2 + \left(\frac{u(n_2)}{n_2}\right)^2}$ and for a ternary system, $u(x_1) = x_1\sqrt{(1 - x_1)^2\left(\frac{u(n_1)}{n_1}\right)^2 + x_2^2\left(\frac{u(n_2)}{n_2}\right)^2 + x_3^2\left(\frac{u(n_3)}{n_3}\right)^2}$

Concerning the solvent, the uncertainty on mole solvent is given by Supplementary Equation (14). U(l) is the uncertainty on piston displacement. We consider that the uncertainty on variable volume cell diameter is negligible.

$$u(n_{solvent}) = n_{solvent}\sqrt{\left(\frac{u(l)}{l}\right)^2 + \left(\frac{u(\rho_{solvent})}{\rho_{solvent}}\right)^2} \qquad (14)$$

In case of one solute, Supplementary Equation (15) should be considered.

$$u(n^L)^2 = u(n^T)^2 + u(n^V)^2 \qquad (15)$$

Concerning the total mole number introduced, Eqs. (16-19) are used.

$$u(n^T)^2 = u(n^{before})^2 + u(n^{after})^2 \qquad (16)$$

$$u(n^{before})^2 = n^{before}\sqrt{\left(\frac{u(V^{press})}{V^{press}}\right)^2 + \left(\frac{u(\rho^{before})}{\rho^{before}}\right)^2} \quad (17)$$

$$u(n^{after})^2 = n^{after}\sqrt{\left(\frac{u(V^{press})}{V^{press}}\right)^2 + \left(\frac{u(\rho^{after})}{\rho^{after}}\right)^2} \quad (18)$$

$$u(n^{T})^2 = \left(n^{before}\sqrt{\left(\frac{u(V^{press})}{V^{press}}\right)^2 + \left(\frac{u(\rho^{before})}{\rho^{before}}\right)^2}\right)^2 + \left(n^{after}\sqrt{\left(\frac{u(V^{press})}{V^{press}}\right)^2 + \left(\frac{u(\rho^{after})}{\rho^{after}}\right)^2}\right)^2 \quad (19)$$

It is important to note that the uncertainties of previous equilibrium data. The link between uncertainty on density and temperature and pressure is given by Supplementary Equation (20). The derivatives are obtained by using REFPROP V10.0.

$$u(\rho)^2 = \left(\frac{\partial\rho}{\partial T}\right)_P^2 u(T)^2 + \left(\frac{\partial\rho}{\partial P}\right)_T^2 u(P)^2 \quad (20)$$

Concerning the total mole number in the vapor phase, we have to consider Eqs. (21 and 22).

$$u(n^V) \quad = n^V\sqrt{\left(\frac{u(\rho^V)}{\rho^V}\right)^2 + \left(\frac{u(V^V)}{V^V}\right)^2} \quad (21)$$

$$u(\rho)^2 = \left(\frac{\partial\rho}{\partial T}\right)_P^2 u(T)^2 + \left(\frac{\partial\rho}{\partial P}\right)_T^2 u(P)^2 \quad (22)$$

Or $P_{Solute} = P_{cell} - P_{solvent}^{sat}$ so we can consider that uncertainty on P_{solute} is identical to the uncertainty on total pressure. Uncertainty on vapor volume is given by Supplementary Equation (23).

$$u(V^V)^2 = u(V^T)^2 + u(V^L)^2 \quad (23)$$

Uncertainty on total volume is given by the volume calibration. Concerning the volume of liquid phase, we have two cases.

1. No variation of volume (Supplementary Equation (24)).

$$u(V^L) \quad = V^L\sqrt{\left(\frac{u(n_{solvent})}{n_{solvent}}\right)^2 + \left(\frac{u\left(\rho(T_{cell})\right)}{\rho(T_{cell})}\right)^2} \qquad (24)$$

2. Measurement of the length of liquid (Supplementary Equation (25).

$$u(V^L)^2 = \left(\frac{u\left(h_{liq}\right)}{h_{liq}}\right)^2 + 2\left(\frac{u(r_{cell})}{r_{cell}}\right)^2 \qquad (25)$$

$u(V^T)$ is known from the calibration of the cell, i.e. $5\cdot10^{-8}$ m^3.

In case of more than one solute, uncertainty on composition have to be taken into account (Supplementary Equation (26))..

$$u(n_i^L)^2 = (z_i n^T)^2\left(\left(\frac{u(z_i)}{z_i}\right)^2 + \left(\frac{u(n^T)}{n^T}\right)^2\right) + (y_i n^V)^2\left(\left(\frac{u(y_i)}{y_i}\right)^2 + \left(\frac{u(n^V)}{n^V}\right)^2\right) \qquad (26)$$

Concerning the total mole number in the vapor phase, its uncertainty is given by Supplementary Equation 21. For the molar density, Supplementary Equation (27) will be considered.

$$u(\rho)^2 = \left(\frac{\partial\rho}{\partial T}\right)_P^2 u(T)^2 + \left(\frac{\partial\rho}{\partial P}\right)_T^2 u(P)^2 + \sum\left(\frac{\partial\rho}{\partial y_i}\right)_T^2 u(y_i)^2 \qquad (27)$$

Or $P_{Solute} = P_{cell} - P_{solvent}^{sat}$ so we can consider that uncertainty on P_{solute} is identical to the uncertainty on total pressure. Uncertainty on vapor volume is given by Supplementary Equation (23).

Molecular Dynamics Simulations

The total energies of the aqueous amine solvents, and solvated CO_2 and OH^- or HCO_3^- were obtained with classical molecular mechanics (MM) dynamics simulations[4] using the large-scale Atomic/Molecular Massively Parallel Simulator software (LAMMPS)[5] together with the Extended Polymer Consistent Force Field (PCFF+)[6-9] as implemented in *MedeA* 3.1.[10] The non-bonded energy terms were truncated beyond a cutoff distance of 9.5 Å. Beyond this distance, the Coulomb interactions were corrected with the particle-particle-particle-mesh (PPPM)[11] method, and the Van der Waals interactions with a tail correction.[12] The Nosé-Hoover thermostat[13] and a barostat[4] were used to control the temperature and pressure, respectively. The Newtonian equation of motion[14] was integrated using a time step of 0.25 fs. The numbers of water and amine molecules in the atomistic models reflect the experimental concentration of 13 mol% amine for the aqueous amine solvents: each simulation, cells contain 25 amine and 168 water molecules. The initial configurations were generated with an imposed density of 0.7 g L^{-1} under periodic boundary conditions using the Amorphous Materials Builder module,[10] which is based on a Monte Carlo approach sampling the different degrees of freedom of the component species to generate realistic configurations of atomistic models. The protocol of equilibration and preparation of these systems was subsequently as follow:

i) The systems were equilibrated at T= 363 K for 1.5 ns in the isothermal-isochoric (NVT) ensemble.

ii) They were then relaxed in the isothermal-isobaric (NPT) ensemble at P=1 atm with a fluctuating temperature from 363 to 313.15 K for 1 ns.

iii) They were then relaxed for 0.5 ns in the NPT ensemble at P=1 atm and T=313.15 K.

iv) An NPT simulation at P=1 atm and T=313.15 K for 5 ns was performed to determine the average density of each system after the equilibration procedure. This computed average density is also used to determine the cell volume of each aqueous amine system and consequently the amine's concentration.

Following this equilibration protocol, the final structure was collected and duplicated: in the first duplicated system, OH^- and CO_2 were inserted in the cell, and in the second, HCO_3^- was added. OH^-, CO_2, and HCO_3^- were placed in the cells to be surrounded with water molecules. The densities of the two systems are set to the value obtained after the 5 ns NPT simulation of aqueous amine solvent in the absence of OH^- and CO_2, or HCO_3^- (step iv above). As OH^-+CO_2 have the same mass as HCO_3^-, the volumes and densities of the two aqueous amine cells, be it with OH^-+CO_2 or HCO_3^- periodic models, are identical. The total energies of the two aqueous amine solvent systems were evaluated from simulations in the NVT ensemble at T=313 K. The energies were sampled over a series of runs. First, 1 and 3 ns runs were done followed by 8 batches of 5 ns NVT runs. The average final energies are the arithmetic simulated-time weighted averages. In some exceptional cases, extreme low or high energy values were discarded in the arithmetic average depending on their deviation with respect to the average, resulting in a minimum sampling time of 30 ns and up to a maximum of 44 ns. A value was considered extreme when its deviation was more than twice the standard deviation with respect to the average value.

Supplementary References

1. Mansouri, K., Grulke, C. M., Judson, R. S. & Williams, A. J. OPERA models for predicting physicochemical properties and environmental fate endpoints. *J. Cheminformatics* **10**, 10 (2018).

2. Kunz, O., Wagner, W. The GERG-2008 wide-range equation of state for natural gases and other mixtures: an expansion of GERG-2004. *Journal of chemical & engineering data*, **57**, 11, (2012).

3. Lemmon, E.; Bell, I. H.; Huber, M.; McLinden, M., NIST Standard Reference Database 23: Reference Fluid Thermodynamic and Transport Properties-REFPROP, Version 10.0, National Institute of Standards and Technology. 2018. *URL http://www.nist.gov/srd/nist23.cfm* 2018.

4. Allen, M. P.; Tildesley, D. J. (1987). *Computer simulation of liquids*. Oxford university press.

5. Plimpton, S. Fast parallel algorithms for short-range molecular dynamics. *Journal of computational physics*, **117**(1), 1-19, (1995).

6. Sun, H., Mumby, S. J., Maple, J. R., Hagler, A. T. An ab initio CFF93 all-atom force field for polycarbonates. *Journal of the American Chemical society*, **116**(7), 2978-2987, (1994).

7. Yiannourakou, M., Ungerer, P., Leblanc, B., Rozanska, X., Saxe, P., Vidal-Gilbert, S., Montel, F. Molecular simulation of adsorption in microporous materials. *Oil & Gas Science and Technology–Revue d'IFP Energies Nouvelles*, **68**(6), 977-994 (2013).

8. Ungerer, P., Rigby, D., Leblanc, B., Yiannourakou, M. Sensitivity of the aggregation behavior of asphaltenes to molecular weight and structure using molecular dynamics. *Molecular Simulation*, **40**(1-3), 115-122 (2014).

9. Rozanska, X., Ungerer, P., Leblanc, B., Saxe, P., Wimmer, E. Automatic and Systematic Atomistic Simulations in the MedeA® Software Environment: Application to EU-REACH. *Oil & Gas Science and Technology–Revue d'IFP Energies nouvelles*, **70**(3), 405-417 (2015).

10. *MedeA*: Materials Exploration and Design Analysis. Copyright © 1998-2020 Materials Design, Inc. Version 3.1.

11. Luty, B. A., Davis, M. E., Tironi, I. G., Van Gunsteren, W. F. A comparison of particle-particle, particle-mesh and Ewald methods for calculating electrostatic interactions in periodic molecular systems. *Molecular Simulation*, **14**(1), 11-20 (1994).

12. Daura, X., Mark, A. E., Van Gunsteren, W. F. Parametrization of aliphatic CH_n united atoms of GROMOS96 force field. *Journal of computational chemistry*, **19**(5), 535-547 (1998).

13. Evans, D. J., Holian, B. L. The nose–hoover thermostat. *The Journal of chemical physics*, **83**(8), 4069-4074 (1985).

14. Frenkel, D.; Smit, B. (2001). *Understanding molecular simulation: from algorithms to applications* (Vol. 1). Elsevier.
